# Phenethyl Isothiocyanate Enhances the Cytotoxic Effects of PARP Inhibitors in High-Grade Serous Ovarian Cancer Cells

**DOI:** 10.3389/fonc.2021.812264

**Published:** 2022-01-26

**Authors:** Yaxun Jia, Min Wang, Xiaolin Sang, Pixu Liu, Jingchun Gao, Kui Jiang, Hailing Cheng

**Affiliations:** ^1^ Cancer Institute, Dalian Key Laboratory of Molecular Targeted Cancer Therapy, The Second Hospital of Dalian Medical University, Dalian, China; ^2^ Institute of Cancer Stem Cell, Dalian Medical University, Dalian, China; ^3^ Department of Obstetrics and Gynecology, The First Hospital of Dalian Medical University, Dalian, China; ^4^ Department of Medical Oncology, The Second Hospital of Dalian Medical University, Dalian, China

**Keywords:** BMN 673, high-grade serous ovarian cancer, PARP inhibitor, phenethyl isothiocyanate, reactive oxygen species

## Abstract

While PARP inhibitor (PARPi) therapies have shown promising results in the treatment of high-grade serous ovarian cancer (HGSOC) harboring homologous recombination deficiencies, primary resistance to PARPi frequently occurs and even initial responders may eventually become resistant. Therefore, the development of novel effective combinatorial strategies to treat HGSOC is urgently needed. Here, we report that H_2_O_2_-induced oxidative stress sensitized HGSOC cells to PARPi BMN 673. Furthermore, Phenethyl isothiocyanate (PEITC) as a ROS-inducing agent significantly enhanced the cytotoxic effects of BMN 673. Mechanistically, combined use of PEITC and BMN 673 resulted in ROS overproduction and accumulation, enhanced DNA damage, G2/M arrest and apoptosis, all of which were significantly reversed by the ROS scavenger *N*-Acetyl-*L*-cysteine. We also showed that while PEITC did not further enhance the ability of BMN 673 on PARP1 trapping in HGSOC cells, the therapeutic effects of the PEITC/BMN 673 combination were at least in part dependent on the presence of PARP1. Importantly, the PEITC/BMN 673 combination potently abrogated the growth of HGSOC tumor spheroids and patient-derived organoid models of HGSOC and cervical cancer. Our findings provide a basis for further investigation of the utility of PARPi combination regimen in HGSOC and cervical cancer through ROS-mediated mechanisms.

## Introduction

High-grade serous ovarian cancer (HGSOC) is among the most common and aggressive histologic subtypes and is associated with poor survival (1, 2). Approximately 50% of HGSOC harbor defects in the homologous recombination repair (HRR) pathways (3). Poly (ADP-ribose) polymerase 1/2 (PARP1/2) is required to repair DNA single-strand breaks, and PARP1 also involved in the repair of DNA replication fork damage and double-strand breaks (4). A substantial body of preclinical and clinical studies have demonstrated that inhibition of PARP1 induces synthetic lethality in HGSOC with HRR deficiencies caused by deleterious *BRCA1/2* mutations or BRCAness (5–7). PARP inhibitor (PARPi)-based therapies are now a standard of care for HGSOC patients. Despite PARPi therapies have shown encouraging results, HGSOC tumors frequently become resistant. Therefore, the development of effective combinatorial strategies to treat this hardly curable disease is urgently needed.

PARPis also cause cytotoxicity by trapping PARP1 at sites of DNA damage (4), another mechanism of action for PARP inhibitors. While all four PARPis including Olaparib, Rucaparib, Niraparib and Talazoparib that have been currently used in the clinic are PARP1/2 catalytic inhibitors, the single-agent cytotoxicity of different PARPi correlates with their effectiveness in trapping PARP1/2, with Talazoparib (BMN 673) exhibiting the superior potency (4). In addition to synthetic lethality of PARP inhibition and HRR deficiencies, additional cytotoxic mechanisms of action underlying PARPi-based combinations have also been reported, including PARP1 trapping (*e.g.* PARPi plus Temozolomide) alone or with concurrent catalytic inhibition of target enzymes (*e.g.* PARPi plus 5-azacytidine) (8–11).

Increased reactive oxygen species (ROS) production in cancer cells is often coupled to redox adaptation that promotes cell growth and survival (12, 13). As excessive amounts of ROS may cause oxidative damage and cell death, cancer cells with increased oxidative stress are likely to be more vulnerable to further ROS insults. Targeting cancer cells with pharmacological agents with pro-oxidant activities by increasing ROS generation or disabling the anti-oxidation system might be an effective strategy to improve therapeutic response and/or overcome drug resistance (12). PARP1 plays an important role in the repair of ROS-induced oxidative DNA damage (14–16). It has been shown that inhibition of PARP1 sensitizes cancer cells to oxidative stress, suggesting a potential therapeutic strategy to kill cancer cells by combining PARPis and pro-oxidant agents. The current study aims to investigate whether phenethyl isothiocyanate (PEITC), a known ROS-generating agent (17–19), enhances the cytotoxic effects of PARPis in HGSOC and cervical cancer. Our findings may provide a basis for further exploration of the utility of PARPi combination regimen in HGSOC and cervical cancer through ROS-mediated mechanisms.

## Material and Methods

### Cell Culture and Plasmids

High-grade serous ovarian cancer (HGSOC) cell lines OVSAHO (RRID: CVCL_3114), SNU119 (RRID: CVCL_5014) and COV362 (RRID: CVCL_2420) were acquired from Otwo Biotech (China). OVCAR4 (RRID: CVCL_1627) was provided by Beijing Zhongke Quality Inspection Biotechnology Co., Ltd. Mall Branch. Cervical cancer cell lines CaSki (RRID: CVCL_1100) and ME180 (RRID: CVCL_1401) were obtained from the American Type Culture Collection (ATCC). Cells were maintained in culture media (OVSAHO in Dulbecco’s Modified Eagle Medium; SNU119, COV362, OVCAR4 and CaSki in RPMI-1640 Medium; ME180 in mcCoy’s 5A (modified) medium) supplemented with 10% fetal bovine serum and 1% penicillin/streptomycin at 37°C and 5% CO_2_. Lentiviral shRNA vector system against PARP1 was purchased from Dhamarcon (USA). PARP inhibitors Talazoparib (BMN 673), Olaparib (AZD2281) and Veliparib (ABT-888) were purchased from Chemexpress (China). Hydrogen peroxide (H_2_O_2_), Phenethyl isothiocyanate (PEITC) and *N*-Acetyl-*L*-cysteine (NAC) were obtained from Sigma (USA).

### Patient Information

We present the cases of a 67-year-old patient with newly diagnosed primary high-grade serous ovarian cancer without family history (OVC_13) and a 41-year-old patient with newly diagnosed primary cervical squamous cell carcinoma (CC_4). Tumor tissue and drainage of ascites acquisition were performed under Institutional Review Board protocols approved by the Second Hospital of Dalian Medical University. Consents were obtained from all patients participating in this project and subjected to withdrawal at any time. The study methodologies conformed to the standards set by the Declaration of Helsinki.

### Clonogenic Assay and Determination of Drug Synergy

HGSOC cells (OVSAHO, SNU119 and COV362: 2000 cells/well; OVCAR4: 1000 cells/well) were seeded on plates and cultured for 24 hours before exposure to drug treatment. Fresh media with or without drugs were replaced every 3 days. At the end point, cells were washed with phosphate-buffered saline and subsequently stained with 0.5% crystal violet. The optical absorbance of the bound crystal violet dissolved in 50% acetic acid was measured at 590 nm by xMark Microplate Spectrophotometer (Bio-Rad Laboratories, USA). The synergy effect was calculated by the Chou-Talalay method to calculate the combination index (CI) (20).

### Flow Cytometry Analysis

For cell apoptosis assay, the Annexin V/PI Apoptosis Detection kit (AD-10, Dojindo Molecular Technologies, Japan) was used according to the manufacturer’s protocol.

For intracellular ROS detection, Reactive Oxygen Species (ROS) Detection Reagents (Invitrogen, #D399) was used according to the manufacturer’s protocol.

For cell cycle analysis, harvested cells were fixed with 75% ethanol overnight followed by staining with a phosphate-buffered saline (PBS) solution containing propidium iodide (50 μg/ml, Sigma) and 100 μg/ml DNase-free RNase A (Sigma). After 30 minutes of incubation, the samples were washed and resuspended in PBS with 0.5% FBS. FACS analyses were performed on a BD FACSCanto™ II (BD Biosciences, USA).

### Western Blot Analysis

Western blot experiments were conducted as described previously (21). Cell lysates were prepared using ice-cold lysis buffer supplemented with protease/phosphatase inhibitors (Roche, Switzerland). The blots were probed with primary antibodies were used: Cleaved PARP (Cell signaling technology, CST, #9541), PARP (CST, #9542), γH2AX (CST, #2577), Poly/Mono-ADP Ribose (CST, #83732), Histone H3 (Proteintech, #17168-1-AP) and Vinculin (Sigma Aldrich, V9131). Western blots were imaged using Odyssey Infrared Imaging System (Li-COR Biosciences, USA).

### Cellular Trapping Assays

Chromatin extraction was performed as described previously (22). Samples were normalized for concentration. Protein binding in the chromatin fraction was assessed by Western blot.

### Three-Dimensional Sphere Assay

The three-dimensional sphere culture experiment was carried out as described previously (23). Briefly, cells were seeded on plates pre-coated with Matrigel (BD Biosciences, USA) and grown in culture medium supplemented with 2% FBS and 2% Matrigel and allowed to grow for 1 day before drug exposure. Fresh medium containing 2% FBS and Matrigel was replaced every 3 days. The 3D structures were imaged by an inverted phase-contrast microscope (Leica Microsystems, Germany) and scored according to 3D structure integrity. Over 100 structures were scored for each condition.

### Organoid Culture and Viability Assay

Organoids were cultured as previously described (24). Briefly, surgically removed cervical tumors were cut into small pieces of tumor tissue. Fresh ascites from ovarian cancer patients was centrifuged to obtain cell pellets. Tumor tissues or cell pellets were then digested with Collagenase Type II (Gibco, USA) followed by the treatment with RBC Lysis Buffer (Bio-Legend, USA). After centrifugation, tissues/cell pellets were suspended in AdDF+++ [Advanced DMEM/F12 containing 1% HEPES (Gibco, USA), 1 x GlutaMAX (Gibco, USA) and antibiotics] and pipetted repeatedly with a syringe, filtered through a 100 μm filter. The isolated cells were mixed with Growth Factor-reduced Matrigel (BD Biosciences, USA) and dropped on 24-well plates. On Matrigel stabilization, the organoid culture medium [AdDF+++ containing 10 ng/ml Noggin (PeproTech, USA), 10 ng/ml Rspo1 (PeproTech, USA), 1.25 mM N-Acetylcysteine (Sigma, USA), 10 mM Nicotinamide (Sigma, USA), 0.5 μM TGFβ Receptor inhibitor A83-01 (Sigma, USA), 10 ng/ml FGF10 (PeproTech, USA), 37.5 ng/ml Heregulin β-1 (PeproTech, USA), 5 μM RhoK inhibitor Y-27632 (AbMole Bioscience, USA), 5 ng/ml EGF (PeproTech, USA), 10 μM Forskolin (Bio-Techne, USA), 500 ng/ml Hydrocortisone (Sigma, USA), 100 nM β-Estradiol (Sigma, USA), 2% B27 supplement (Gibco, USA), and 0.2% Primocin (*In vivo*Gen, USA)] was added and the plates were transferred to humidified 37°C/5% CO_2_ incubators. For passaging, organoids were digested with TrypLE Express Enzyme (Thermo Fisher Scientific, USA) and re-inoculated to form new organoids. The organoid culture medium was changed every 3 days and passaged every 1 to 4 weeks. Organoid structures were imaged by an inverted phase-contrast microscope (Leica Microsystems, Germany).

For organoid viability assay, organoids were dissociated and cultured in 96-well plates followed exposure to the organoid culture medium containing PEITC and/or BMN 673. The organoid culture medium was changed every 3 days. The ATP levels were measured with CellTiter-Glo^®^ 3D Reagent (Promega, USA) according to the manufacturer’s instructions. Luminescence signals were measured using a SpectraMax microplate reader (Molecular Devices, Austria).

### Statistical Analysis

The statistical analyses of the data were performed using GraphPad Prism and specified in the Figure legends. *p <*0.05 was considered to be statistically significant.

## Results

### H_2_O_2_ Treatment Sensitizes HGSOC Cells to BMN 673

**Figure 1:**
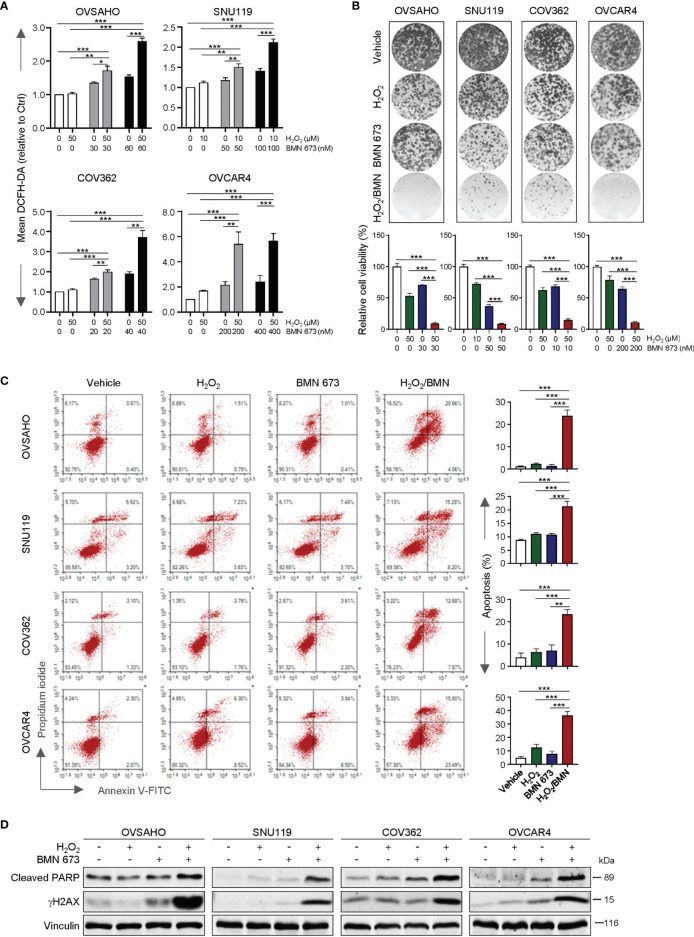
H_2_O_2_ enhanced the antitumor activity of BMN 673 in the HGSOC cells. **(A)** FACS analysis of ROS levels in the HGSOC cell lines treated with BMN 673 in the presence or absence of H_2_O_2_ for 48 hours. **(B)** Clonogenic survival assay of the HGSOC cells with drug treatments as indicated for 6-8 days. At the endpoint, the plates were fixed and stained with crystal violet. Representative images of the plates from each group are shown. **(C)** Apoptosis levels in the cells as in **(B)** were determined by Annexin V/PI staining and FACS analysis. Data are shown as mean ± S.D. *p < 0.05; **p < 0.01; ***p < 0.001 (Student’s *t*-test). **(D)** Western blot analysis of Cleaved PARP and γH2AX in the cells treated as in **(B)**. Vinculin was used as a loading control.

We first assessed whether the PARP inhibitor BMN 673 would affect the intracellular redox status in a panel of HGSOC cell lines, including OVASHO, SNU119, COV362 and OVCAR4. BMN 673-treated cells contained little to moderate increase in intracellular ROS levels when compared with the vehicle-treated cells (**Figure 1A**). Upon treatment with H_2_O_2_, BMN 673-treated cells exhibited a pronounced elevation of ROS levels across all four cell lines examined (**Figure 1A**). Strikingly, chronic exposure to H_2_O_2_ and BMN 673 in combination dramatically blocked the growth of HGSOC cells (**Figure 1B**). Furthermore, the combination treatment, but not either single-agent, led to a substantial increase in apoptosis as evidenced by FACS analysis of Annexin V/PI-stained cells and western blot analysis of cleaved PARP signals (**Figures 1C, D**). We also observed a substantial induction of DNA damage as indicated by γH2AX signals in the cells treated with H_2_O_2_ and BMN 673 in combination (**Figure 1D**). Together, these data indicate that oxidative stress may sensitize HGSOC cells to BMN 673 treatment.

### PEITC Treatment Sensitized HGSOC Cells to BMN 673 *via* Induction of Excessive ROS Levels

**Figure 2:**
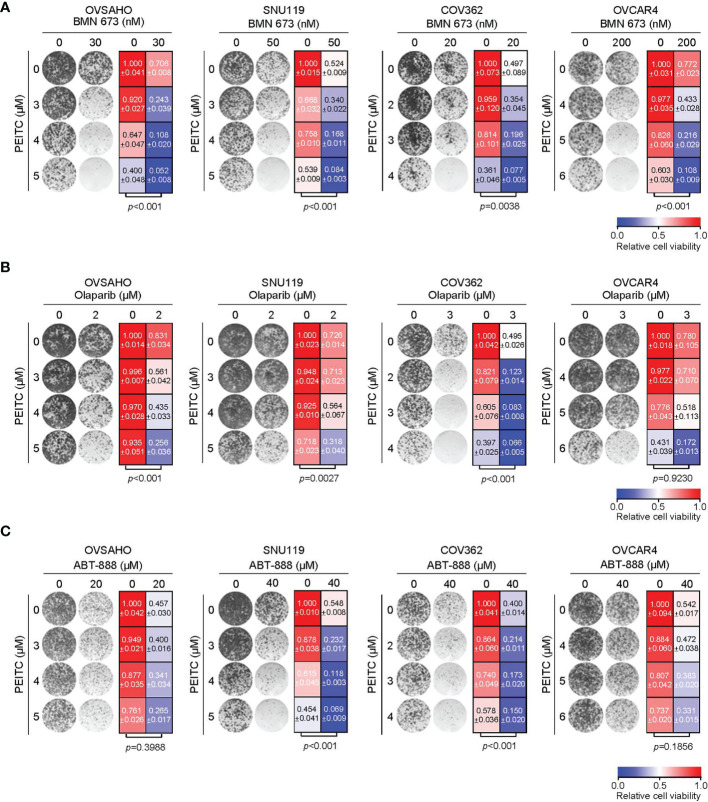
Treatment with PEITC sensitized the HGSOC cells to PARP inhibitors. Representative images (left) and quantification (right) of long-term clonogenic assay with the HGSOC cells [**(A)**, PEITC/BMN 673; **(B)**, PEITC/Olaparib; **(C)**, PEITC/ABT-888] treated as indicated. Data are shown as mean ± S.D. *p* values were shown as indicated (Two-way ANOVA).

We next investigated whether the ROS inducer PEITC may sensitize HGSOC cells to PARP inhibitors. In all four HGSOC cell lines examined, combined use of PEITC and BMN 673 demonstrated synergistic cytotoxic effects as assessed by the median-drug effect analysis (**Figure S1**). Concordantly, the long-term clonogenic survival assays revealed that PEITC treatment significantly enhanced the cytotoxic effect of PARP inhibitor BMN 673 (**Figures 2A** and **S2**). Similar observations were seen with the combined use of PEITC and Olaparib, another PARP inhibitor, in OVSAHO, SNU119 and COV362 but not OVCAR4 cells (**Figure 2B**). In contrast, PEITC sensitized two (SNU119 and COV362) but not the other HGSOC cell lines (OVSAHO and OVCAR4) to ABT-888 (Veliparib), an inhibitor with weak PARP1 trapping activity (4) (**Figure 2C**).

**Figure 3:**
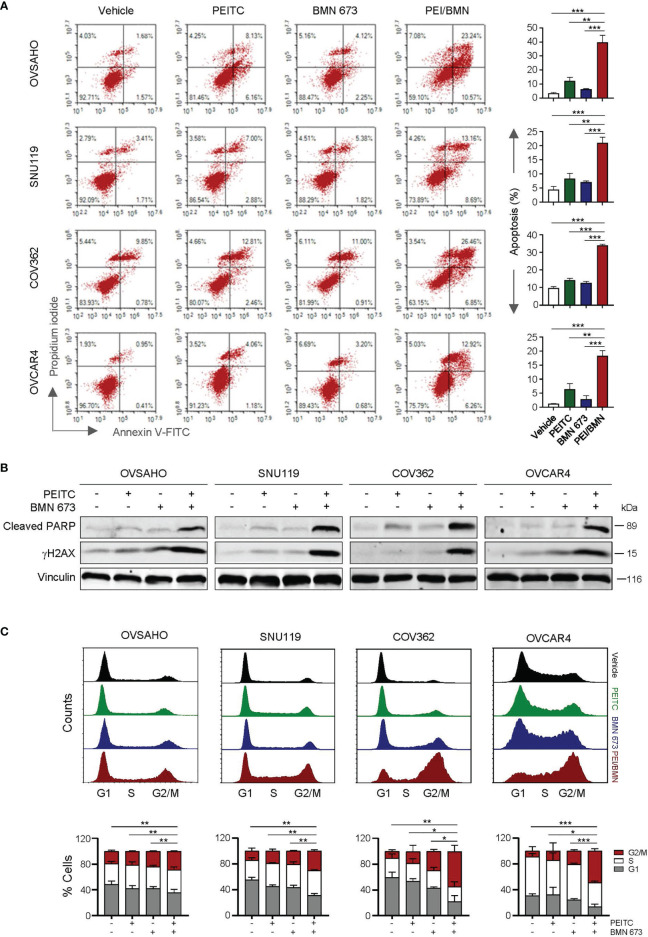
The combination of PEITC and BMN 673 induced DNA damage, apoptosis and G2/M arrest in the HGSOC cells. **(A)** Apoptosis levels in the cells treated as indicated for 48 hours were determined by Annexin V/PI staining and FACS analysis. **(B)** Western blot analysis of Cleaved PARP and γH2AX in the cells. Vinculin was used as a loading control. **(C)** Cell cycle analysis of the HGSOC cells treated as indicated for 24 hours was determined by PI staining and FACS analysis. Data are shown as mean ± S.D. The statistical differences in the percentage of G2/M cells were indicated. *p < 0.05; **p < 0.01; ***p < 0.001 (Student’s *t*-test).

Similar to the effects of oxidative stress induced by H_2_O_2_, treatment with the ROS inducer PEITC also led to a significant increase in apoptotic cell death as well as DNA damage in the cells simultaneously exposed to BMN 673 (**Figures 3A, B**). In addition, the cell cycle analysis showed that while BMN 673 induced G2/M arrest, the drug combination with PEITC led to a further substantial accumulation (**Figure 3C**). Together, these data suggest that PEITC may synergize with BMN 673 to induce DNA damage, leading to cell cycle arrest at G2/M and apoptotic cell death.

**Figure 4:**
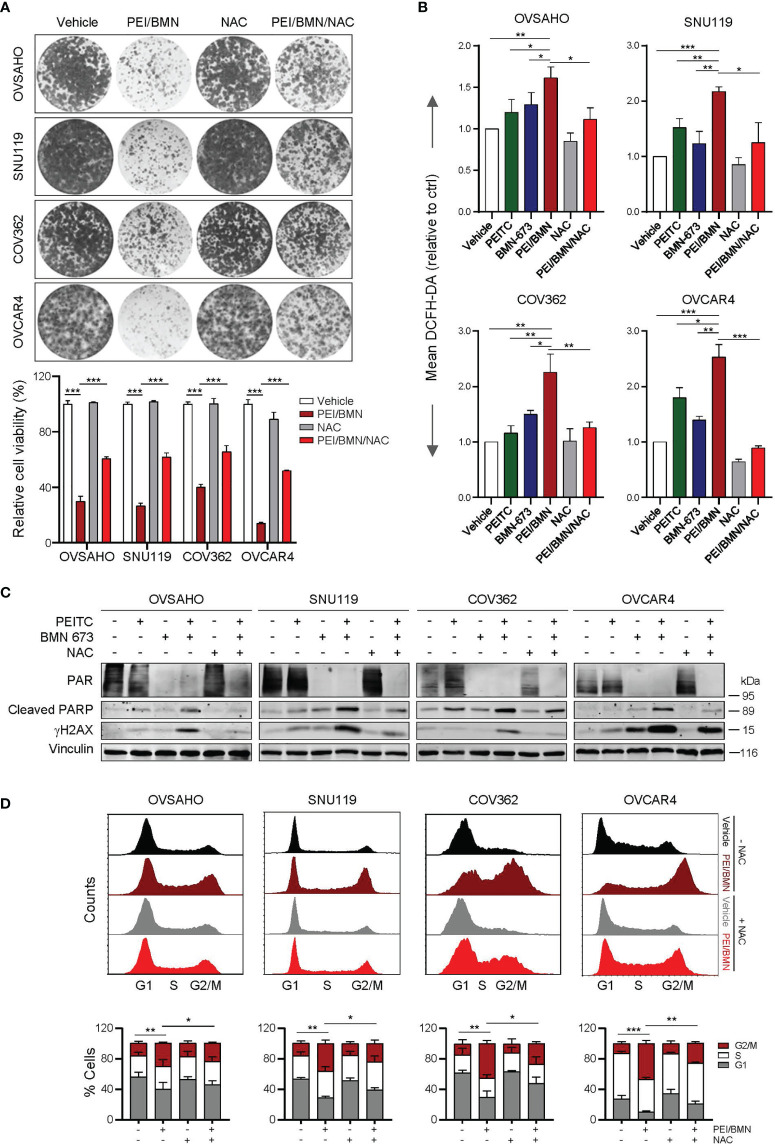
Treatment with NAC significantly reversed the synergistic effects of the PEITC/BMN 673 combination in the HGSOC cells. **(A)** Clonogenic survival assay of the HGSOC cells with drug treatments as indicated for 6-8 days. Representative images of the plates from each group were shown. **(B)** FACS analysis of ROS levels in the HGSOC cell lines treated as indicated for 48 hours. **(C)** Western blot analysis of PAR, Cleaved PARP and γH2AX in the cells treated as indicated. Vinculin was used as a loading control. **(D)** Cell cycle analysis of the HGSOC cells treated as indicated for 24 hours was determined by PI staining and FACS analysis. Data are shown as mean ± S.D. The statistical differences in the percentage of G2/M cells were indicated. *p < 0.05; **p < 0.01; ***p < 0.001 (Student’s *t*-test).

To further understand the mechanism underlying the synergistic action of PEITC and BMN 673, we used the ROS scavenger *N*-acetyl-*L*- cysteine (NAC) to assess whether the drug combination-induced cytotoxicity is ROS-dependent. While combined use of PEITC and BMN 673 yielded a marked ROS accumulation, NAC treatment significantly reduced ROS levels and negated the sensitivity of HGSOC cells to the drug combination (**Figures 4A, B**). Further analysis showed that treatment with NAC also led to attenuated effects on DNA damage, apoptosis and G2/M arrest induced by the PEITC/BMN 673 combination (**Figures 4C, D**). Nevertheless, as NAC treatment did not restore the PAR signal (a biomarker for PARP activity) reduced by the PEITC/BMN 673 combination (**Figure 4C**), we argued that ROS may not affect the inhibitory action of BMN 673 on PARP1 activity in these HGSOC cells. Together, these data suggest that PEITC may enhance the cytotoxic effect of PARP inhibitors through inducing excessive oxidative stress in HGSOC cells.

### The PEITC and BMN 673 Combination-Induced Cytotoxicity Is PARP1 Dependent

**Figure 5:**
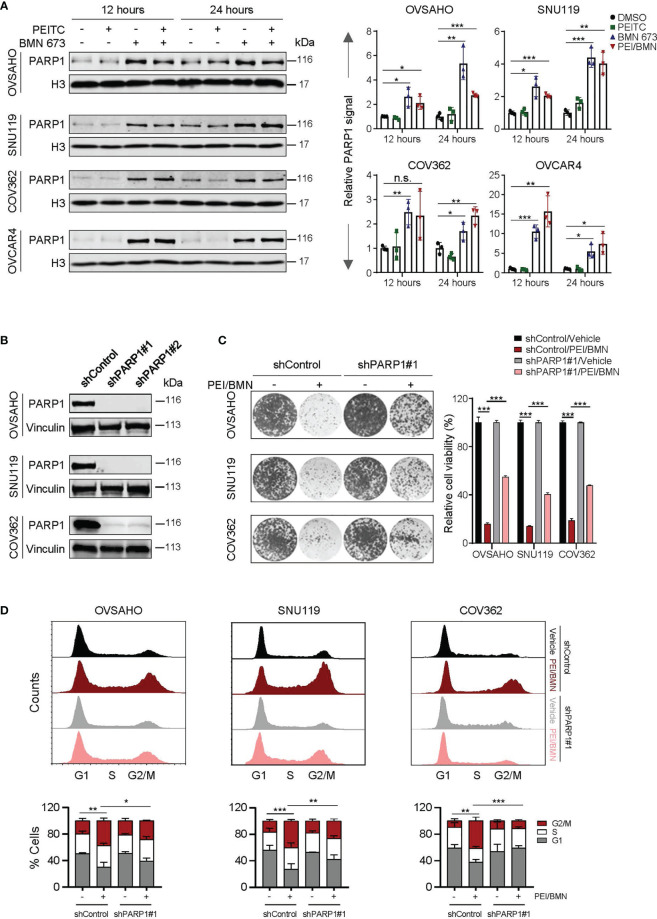
The effects of the PEITC/BMN 673 combination were PARP1-dependent. **(A)** PARP-trapping assay. Western blot analysis of PARP1 in chromatin fractions in the HGSOC cell lines (OVSAHO, SNU119, COV362 and OVCAR4) treated as indicated. Histone 3 was used as a loading control. **(B)** Western blot analysis of PARP1 in the HGSOC cell lines (OVSAHO, SNU119 and COV362) with or without shRNA-mediated PARP1 knockdown. Vinculin was used as a loading control. **(C)** Clonogenic survival assay of the HGSOC cell lines treated with or without the PEITC/BMN 673 combination. **(D)** Cell cycle analysis of the HGSOC cells treated as indicated for 24 hours was determined by PI staining and FACS analysis. Data are shown as mean ± S.D. The statistical differences in the percentage of G2/M cells were indicated. n.s., not significant; *p < 0.05; **p < 0.01; ***p < 0.001 (Student’s *t*-test).

We next wondered whether PEITC-induced oxidative stress may promote PARP1-trapping in HGSOC cells treated with BMN 673. Treatment with the PEITC/BMN 673 combination yielded amplitudes of PARP1-trapping comparable to BMN 673 single-agent treatment in all four HGSOC cell lines examined (**Figure 5A**). As PEITC did not further enhance the chromatin-bound of PARP1, the synergistic action of PEITC and BMN 673 cannot be explained by elevated PARP1 trapping-induced cytotoxicity.

To examine whether the cytotoxicity induced by the PEITC/BMN 673 combination depends on PARP1, we generated the HGSOC cells with shRNA*-*mediated knockdown of PARP1 (**Figure 5B**). In response to the drug combination, the cells with silenced PARP1 expression exhibited significantly reduced drug sensitivity and decreased G2/M arrest (**Figures 5C, D**). Together, these data indicate that while PEITC did not further enhance the ability of BMN 673 on PARP1 trapping in HGSOCs, the therapeutic effects of the PEITC/BMN 673 combination are at least in part dependent on the presence of PARP1.

### The PEITC/PARPi Combination Abrogated the Growth of Tumor Spheroids and Patient-Derived Organoids

**Figure 6:**
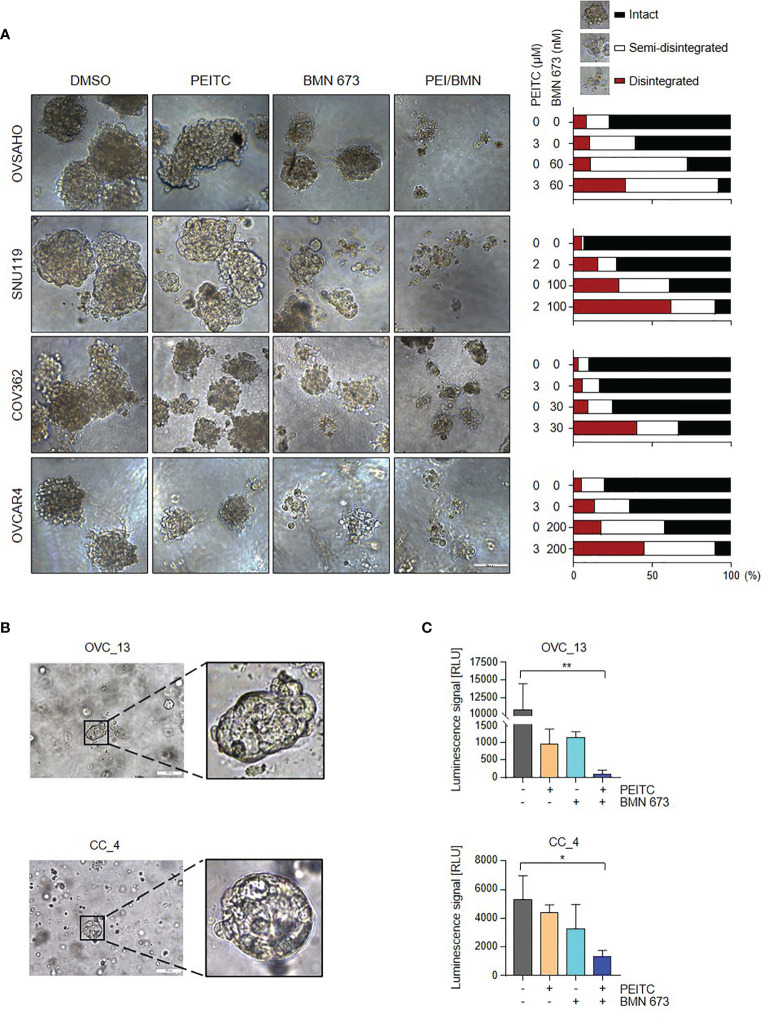
The PEITC/BMN 673 combination significantly abrogated the growth of the 3D HGSOC tumor spheroids and organoids. **(A)** HGSOC cells were cultured in Matrigel and treated with PEITC and BMN 673, either alone or in combination. Representative images (left) and Quantification (right) of scored structures (intact, semi-disintegrated and disintegrated) are shown. Scale bars, 100 μm. **(B)** Representative phase-contrast images of OVC_13 organoid cultured on Day 9 and CC_4 organoid cultured on Day 7 were shown (passage P0). Scale bars, 100 μm. **(C)** ATP levels (Luminescence signal) of organoids treated with PEITC and BMN 673, alone or in combination, were shown. OVC_13: PEITC, 14 μM; BMN 673, 400 nM; 23-day drug treatment. CC_4: PEITC, 7 μM; BMN 673, 800 nM; 24-day drug treatment. Data are shown as mean ± S.D. *p < 0.05; **p < 0.01 (one-way ANOVA followed by Multiple comparisons test).

We next assessed the responses of HGSOC cells to PEITC and/or PARPi in conditions that mimic natural microenvironment *in vivo*. In a three-dimensional (3D) culture model, combined treatment with PEITC and BMN 673 induced massive disintegration of the tumor spheroids in all four HGSOC cell lines examined compared to each single treatment group (**Figure 6A**), suggesting the potential of the PEITC-PARPi combination in the treatment of HGSOC.

Organoid cultures of patient-derived tumors have been demonstrated useful for assessing specific agents for cancer patients (24–27). We have recently established organoid culture models of gynecological cancers. Tumor tissue obtained from consenting patients who underwent surgical removal or drainage of ascites was dissociated and the isolated tumor cells were then suspended in growth factor-reduced Matrigel and cultured in specific organoid culture medium. As shown in **Figure 6B**, we have established two organoid lines derived from the ascites of a high-grade serous ovarian cancer patient and the primary tumor of a cervical squamous cell carcinoma patient without prior treatments, named OVC_13 and CC_4, respectively.

To validate our *in vitro* findings, we employed organoid models to examine the therapeutic effect of the PEITC/BMN 673 combination. After 24 days of drug treatment, we performed a cell viability assay to evaluate organoid drug sensitivity. Indeed, combined use of PEITC and BMN 673 exerted potent therapeutic effects in OVC_13 and, to a lesser extent, CC_4 organoid line (**Figure 6C**). In line with these, the cervical cancer cell lines CaSki and ME180 showed significant response to the drug combination when compared to single-agent treatments *in vitro* (**Figure S3A, B**). Together, these data suggest the potential clinical utility of PEITC/BMN 673 combination in the treatment of HGSOC and cervical cancer.

## Discussion

High-grade serous ovarian cancer is often associated with poor prognosis and high mortality (2). Despite the success of PARP inhibitors in the treatment of this disease, primary resistance frequently occurs and even initial responders may become resistant (6). Effective PARPi-based combinatorial strategies are thus urgently needed to treat this difficult disease. Numerous studies have pointed to targeting of non-oncogene addictions such as oxidative stress that are essential for cancer cell survival as attractive therapeutic approaches (28, 29). PARP1 plays an important role in the repair of oxidative DNA damage (15, 16). The current study aims to assess whether the ROS inducer PEITC may enhance the cell-killing effects of PARP inhibitors in HGSOC cells. Utilizing 2D and 3D culture models of cancer cell lines as well as patient-derived organoid models of HGSOC and cervical cancer, we show that PEITC synergizes with PARP inhibitors to confer cytotoxicity through inducing excessive ROS levels and DNA damage.

Recent studies reveal that PARP inhibitors exert antitumor effects by not only inducing DNA damage but also elevating oxidative stress (5, 14, 30–33). Several natural compounds or inhibitors with the capability to induce ROS levels have been shown to cause synthetic lethality with PARP inhibitors in cancer cells irrespective of *BRCA* or homologous recombination repair status. For example, Berberine, a compound found in many medicinal herbs, confers increased sensitivity to PARP inhibition through inducing oxidative stress and impairing homologous recombination repair in ovarian cancer cells (30). Additionally, induction of oxidative DNA damage by the natural compound Alantaolactone confers synergistic lethality with PARP inhibitor-mediated PARP-trapping activity in prostate cancer cells (31). APR-246, a first-in-class reactivator of mutant p53, synergizes with PARP inhibitors to induce ROS overproduction and apoptosis in p53 mutant non-small cell lung cancer cells (32). In the current study, we investigated the potential therapeutic value of combined use of the ROS inducer PEITC and PARP inhibitors in the treatment of HGSOC and cervical cancer cells.

PEITC, a natural product found in cruciferous vegetables, is capable of inducing ROS production and conferring cytotoxic effects specifically to cancer cells (17–19). Accumulating research articles on the anti-cancer activities of PEITC are available, and several dozens of cancer-related biological targets of PEITC have thus far been identified (18, 19, 34). It is worth noting that PEITC has been shown to inhibit drug transporter proteins such as P-glycoprotein 1 (PgP1), multi-drug resistance protein1 (MRP1) and breast cancer associated protein (BCRP), thus having the potential to improve drug bioavailability (35–38). Indeed, pre-clinical studies have reported the association of improved outcomes and drug combinations of PEITC and conventional chemotherapeutic agents such as docetaxel, doxorubicin and histone deacetylase (HDAC) inhibitors (39–41). PARP inhibitors including Olaparib, Rucaparib and BMN 673 are substrates for drug efflux pump proteins (4, 42, 43), making hyperactivation of drug transporters potential mechanisms of drug resistance to PARP inhibitors. PEITC-mediated inhibition of drug efflux pumps may thus prevent PARP inhibitors from pumping out of the cell and elevate local concentration of drugs. This scenario may account for, at least in part, the synergistic effect of PARPi and PEITC seen in the current study. Our future investigations will also evaluate the therapeutic tolerability of the drug combination using *in vivo* models. On the other hand, as HGSOC represents the most prevalent and aggressive subtype of ovarian cancers that are approved for clinical use of PARP inhibitors, our study purposedly chose ovarian cancer cell line models featuring genomic profiling of HGSOC, including SNU119, OVSAHO, COV362 and OVCAR4 (44). It has been reported that cancer cells with mutated p53 are relatively more sensitive to PEITC than those bearing wild type p53 (45–47). Of note, as majority of HGSOCs including all of the cell lines examined in our study are also p53 mutant (44), our data coincide with the previous reports on the association of PEITC efficacy with p53 mutant cancers.

Our work provides the first evidence that PEITC potentiates the cytotoxic effects of PARP inhibitor BMN 673 in HGSOC cells. Among the three PARP inhibitors used in this study, BMN 673 has the most potent and ABT-888 has the least PARPi activity towards HGSOC cells. Although PEITC treatment failed to yield further enhanced PARP-trapping caused by BMN 673, knockdown of PARP1 significantly attenuated the growth inhibitory effect of BMN 673 and PEITC, indicating that BMN 673-mediated PARP1 trapping activity may contribute, at least in part, to the synergistic lethality induced by the drug combination. Of note, not only Olaparib but also Veliparib (ABT-888) synergizes with PEITC to induce cytotoxicity in some of the HGSOC cell lines, suggesting that inhibition of PARP enzyme activity can be still important to induce synergistic cytotoxicity.

Unlike HGSOC, the pathogenesis of cervical cancer cannot be ascribed to defects in homologous recombination repair pathway (48–50). Nevertheless, the potential of PARPi-based monotherapy and combination therapy in cervical cancer has been under active clinical investigation (51, 52). In the current study, our data suggest that PEITC may enhance the cytotoxic effects of BMN 673 in not only ovarian cancer but also cervical cancer. Organoid models have provided a useful tool that enable patient-specific drug testing and the development of novel therapeutic regimens (24–27). More importantly, we demonstrate that the PEITC/BMN 673 combination exhibits potent therapeutic effect on the growth of HGSOC- and cervical tumor-derived organoids. Together, our work suggests a promising combinatorial strategy in which the ROS inducer PEITC synergizes with PARP inhibitors to induce cytotoxicity in HGSOC and cervical cancer.

## Data Availability Statement

The original contributions presented in the study are included in the article/**Supplementary Material**. Further inquiries can be directed to the corresponding authors.

## Ethics Statement

Institutional Review Board protocols approved by the Second Hospital of Dalian Medical University. The patients/participants provided their written informed consent to participate in this study.

## Author Contributions

HC, PL, KJ, JG, and YJ conceived and designed the study. HC, PL, YJ, and XS wrote the manuscript. YJ performed major experiments, collected, and analyzed data. YJ, MW, and XS performed flow cytometric analysis. All authors contributed and approved the manuscript.

## Funding

This work was supported by the Liaoning Revitalization Talents Program (XLYC2002043 to PL), the Liaoning Provincial Key Research and Development Program (No. 2020JH2/10300049 to PL), Dalian Leading Talents Fund (203598 to PL), the Doctoral Foundation of Liaoning Province (No. 2020-BS-188 to MW), the National Natural Science Foundation of China (No. 81172457 to JG), the Natural Science Foundation of Liaoning Province (No. 20170540234 to KJ) and Science and Technology Innovation Fund of Dalian Department of Science and Technology (2020JJ27SN079 to HC, 2021JJ12SN39 to PL).

## Conflict of Interest

The authors declare that the research was conducted in the absence of any commercial or financial relationships that could be construed as a potential conflict of interest.

## Publisher’s Note

All claims expressed in this article are solely those of the authors and do not necessarily represent those of their affiliated organizations, or those of the publisher, the editors and the reviewers. Any product that may be evaluated in this article, or claim that may be made by its manufacturer, is not guaranteed or endorsed by the publisher.

## Abbreviations

HGSOC, high-grade serous ovarian cancer; PARP, poly (ADP-ribose) polymerase; PARPi, PARP inhibitor; PEITC, phenethyl isothiocyanate; H_2_O_2_, hydrogen peroxide; ROS, reactive oxygen species; NAC, *N*-Acetyl-*L*-cysteine.
