# Supplementary figures and images for: Phenethyl Isothiocyanate Enhances the Cytotoxic Effects of PARP Inhibitors in High-Grade Serous Ovarian Cancer Cells
Source: Front Oncol. 2022 Jan 26;11:812264. doi: 10.3389/fonc.2021.812264 (PMC8825372; doi:10.3389/fonc.2021.812264)

Supplementary Material

# Supplementary Figure


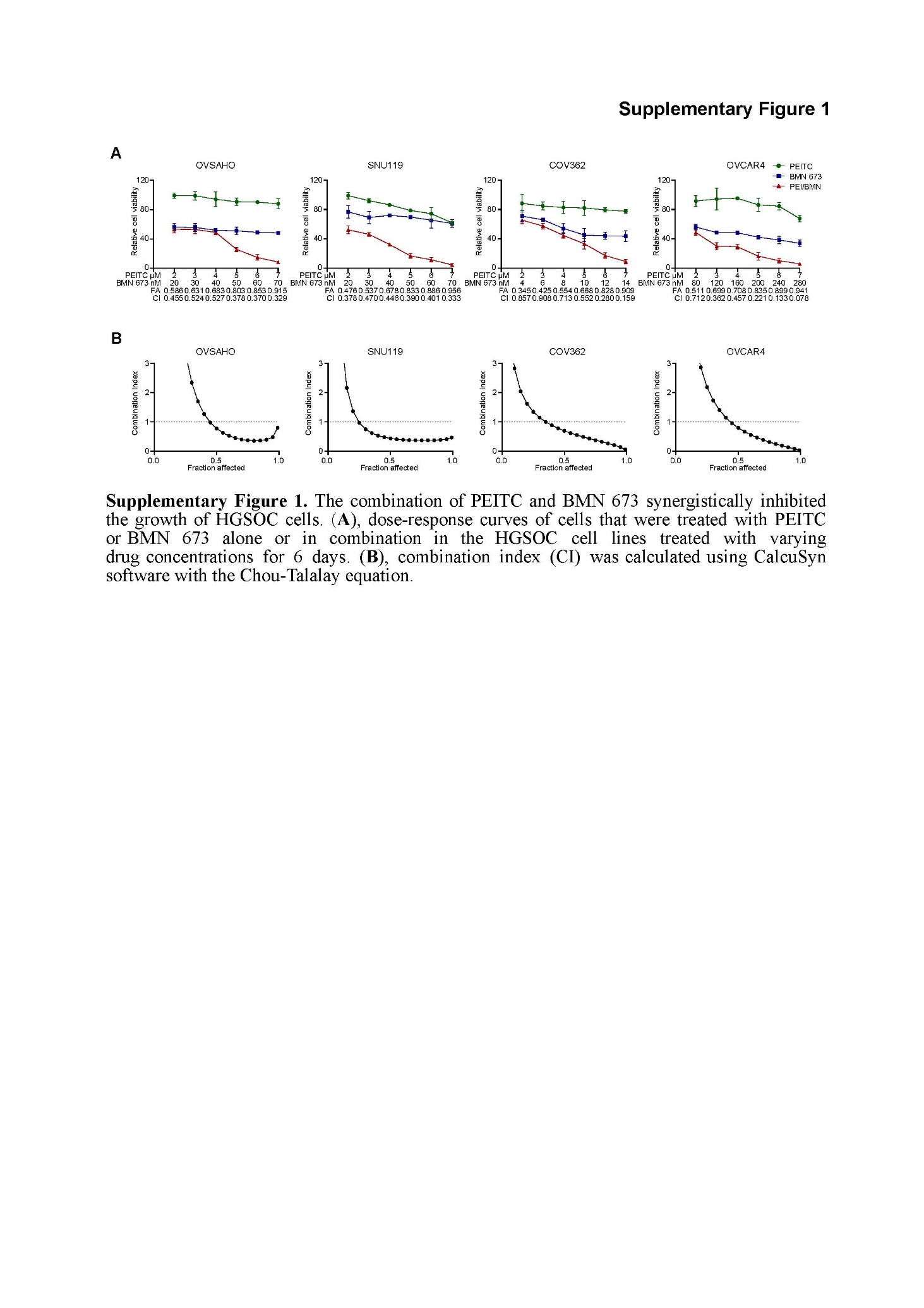


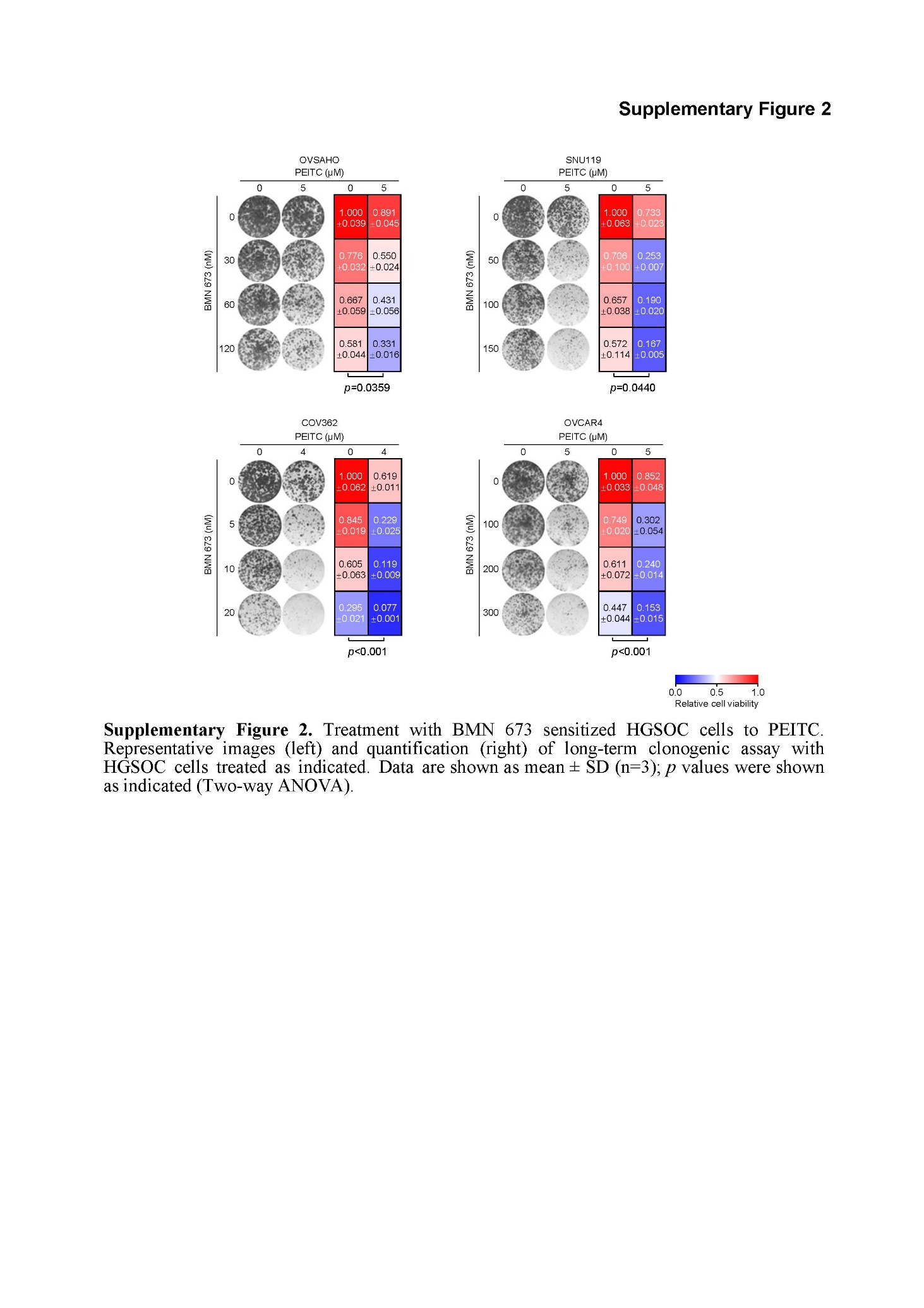

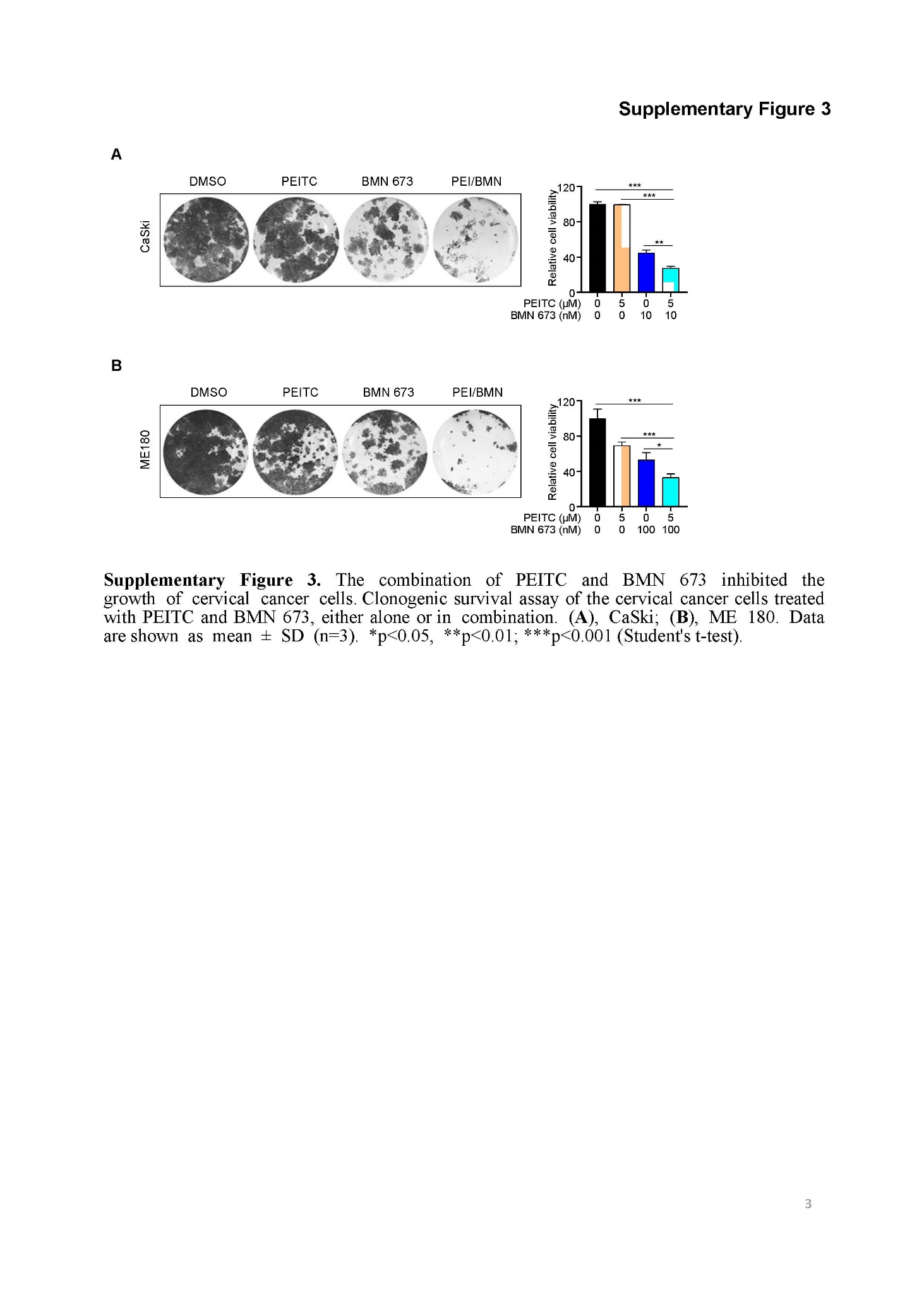

Supplement: Supplementary file 1 [file DataSheet_1.docx]
